# The changing influence of host genetics on the leaf fungal microbiome throughout plant development

**DOI:** 10.1371/journal.pbio.3001748

**Published:** 2022-08-12

**Authors:** Talia L. Karasov, Derek S. Lundberg

**Affiliations:** 1 School of Biological Sciences, University of Utah, Salt Lake City, Utah, United States of America; 2 Swedish University of Agricultural Sciences, Uppsala, Sweden

## Abstract

Host genetics and the environment influence which fungal microbes colonize a plant. This Primer explores a new study in PLOS Biology which finds that the relative influence of these factors changes throughout the development of the biofuel crop switchgrass growing in the field.

## Teaser

Host genetics and the environment influence which fungal microbes colonize a plant. A new study in *PLOS Biology* finds that the relative influence of these factors changes throughout the development of the biofuel crop switchgrass growing in field settings.

The microbes that colonize plants impact plant health, influencing a range of traits including seed production, flowering time, disease susceptibility, and the ability to attract pollinators. Of course, some also cause disease. Together, these microbes comprise the plant’s microbiome. Given the importance of microbes for plant productivity, a central focus in plant biology has become determining and then harnessing factors that control the membership and activity of the plant microbiome. An ultimate goal of this research is to improve agricultural output and sustainability. Crucial to achieving this goal is an understanding of what distinguishes a productive plant microbiome from a diseased microbiome as well as determining which variables influence microbial composition.

Several factors are associated with plant microbiome compositional differences, including host genetics, the environment, and interspecies interactions. Environmental differences clearly play a role [1] as do interspecies interactions [2]. Work over the past 2 decades probed the role of host genetics in microbiome composition, revealing moderate heritability and many loci of small effect that influence composition [3,4]. Time-course experiments also suggested that the leaf microbiome is dynamic, changing throughout the seasons [5,6].

Despite these important findings, we currently lack a predictive understanding of microbiome composition. Both genotype and environment have an influence, but do their relative contributions change during plant development? Are patterns of microbial succession predictable across environments? Furthermore, with rare exceptions, the genetic changes underlying heritable differences in microbiome composition remain unknown.

**Fig 1:**
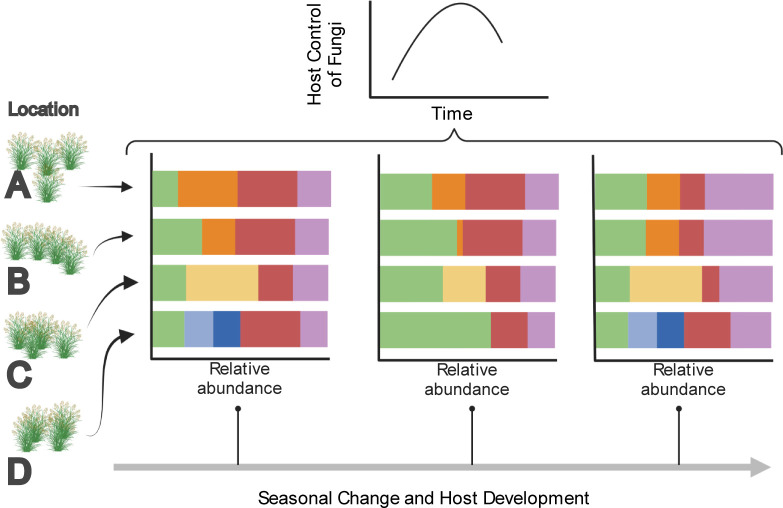
Genetic control of the fungal microbiome changes through time. Schematic of experimental setup and measurements taken. The same switchgrass genotypes were grown at 4 different field sites, and their fungal microbiomes were sequenced repeatedly throughout the growth season. The authors found that the influence of genetics on the fungal community was not static, instead changing over time. Created with BioRender.com.

Switchgrass (*Panicum virgatum*) is a genetically diverse perennial grass native to and distributed throughout North America that is planted in agriculture for feedstock and biomass energy production. A new study published in *PLOS Biology* by Van Wallendael and colleagues investigates factors influencing the fungal leaf microbiome of switchgrass [7] (Fig 1). This research was an impressive logistical feat involving repeated longitudinal measurements of hundreds of plants, including at field sites at different latitudes in the United States of America. Through sequencing and analysis of Internal Transcribed Spacer (ITS) amplicons, the authors found significant contributions of environment and host genetics to fungal microbiome composition and showed that the relative importance of these variables changes dynamically throughout the growing season.

Van Wallendael and colleagues studied variation in fungal microbial composition throughout the year in 2 main experiments. First, they used a panel of 106 switchgrass genotypes sampled at 5 time points in Michigan, and next, they used a panel of 8 switchgrass genotypes replicated in parallel at 4 locations through the USA (Michigan, Missouri, and 2 in Texas). These experiments allowed for the simultaneous assessment of (i) the effect of host genotype on microbiome composition; (ii) the dynamics of microbiome composition throughout the year; and (iii) the consistency of genotypic effects across environments. They found that the collection site explained more than 5 times the variance in microbiome composition than did the genetic subpopulation, though both factors were significant. Strikingly, the association between genetics and composition was not static, instead increasing in strength (Mantel’s R) throughout the growth season until seeds set and then decreasing with the onset of senescence. Because the host genotypes were fully sequenced, the authors were further able to map genetic loci associated with variation in microbiome composition in the large Michigan experiment. Stringent genome-wide association mapping of the association between microbiome composition and plant genotype identified a locus on chromosome 2N with 3 genes in close proximity that are all paralogs of a cysteine-rich receptor-like kinase (RLK) [8]. RLKs comprise a large gene family that encodes signaling proteins, and members of the RLK subfamily cysteine-rich RLKs are involved in responses to abiotic stress and immune induction [9,10]. All 3 genes exhibited expression variation between ecotypes, further suggesting one or more of these genes is involved in compositional shifts between plants.

Several avenues of future research are opened by these findings. The RLKs identified in the association mapping are good candidates for involvement in microbial proliferation. Most of our understanding to date of host genetic control of microbiota has centered around interactions with single microbial species or even strains—for example, the role of plant resistance genes in suppressing specific pathogens. The genetic loci identified here are associated with differences in the multivariate composition of the microbiome and not a single microbe. The genetic pathways underlying a broad-spectrum shift in microbial composition on plant leaves are largely unexplored, partly because of the difficulty in performing a well-controlled microbiome experiment in field conditions with a large enough sample size to distinguish signal from noise. It will be important to manipulate these genes in switchgrass and confirm their role in fungal associations, to identify what molecules these gene products may be detecting, and further to determine if these genes also may affect bacterial communities.

Why genetic control is strongest at the time of seed set is unknown and another interesting avenue for future study. Furthermore, is the change in genetic control due to developmental change of the plant or seasonal environmental shifts? The fact that this successful study was conducted on field grown plants provides a proof of concept that should encourage other researchers to study other plant–microbe interactions in field conditions.

The assembly of a microbiome is complex, involving thousands of interactions between microbes, between microbes and the environment, and between microbes and the host. Due to this complexity, we as a scientific community are unlikely to uncover any single factor that is able to toggle a plant microbiome deterministically between healthy and unhealthy. If we wish to harness the microbiome to improve plant health, we must begin to determine the many rules of interaction, determine their relative importance, and determine how they change over time.
